# Current Sensor Fault Reconstruction for PMSM Drives

**DOI:** 10.3390/s16020178

**Published:** 2016-01-30

**Authors:** Gang Huang, Yi-Ping Luo, Chang-Fan Zhang, Jing He, Yi-Shan Huang

**Affiliations:** 1 School of Traffic and Transportation Engineering, Central South University, Changsha 410075, China; gangder@csu.edu.cn (G.H.); ypluo@csu.edu.cn (Y.-P.L.); 2 College of Electrical and Information Engineering, Hunan University of Technology, Zhuzhou 412007, China; zhangchangfan@263.net; 3 Hunan CSR Times Electric Vehicle Co., Ltd, Zhuzhou 412007, China; huangys@teg.cn

**Keywords:** permanent magnet synchronous motor (PMSM), active flux, sliding mode observers, sensors fault, reconstruction

## Abstract

This paper deals with a current sensor fault reconstruction algorithm for the torque closed-loop drive system of an interior PMSM. First, sensor faults are equated to actuator ones by a new introduced state variable. Then, in αβ coordinates, based on the motor model with active flux linkage, a current observer is constructed with a specific sliding mode equivalent control methodology to eliminate the effects of unknown disturbances, and the phase current sensor faults are reconstructed by means of an adaptive method. Finally, an αβ axis current fault processing module is designed based on the reconstructed value. The feasibility and effectiveness of the proposed method are verified by simulation and experimental tests on the RT-LAB platform.

## 1. Introduction

Permanent magnet synchronous motors (PMSMs) are attractive for electric vehicle and railway traction drive applications thanks to their small volume, light weight, high efficiency, high power density, rugged construction and faster response [1,2]. However, PMSM traction systems are easily influenced by the circumstances of the application environment such as vibration and shock, low and high temperature, humidity and dust, *etc*., which lead to faults that could directly result in deterioration of the torque performance, and even seriously affect the safety of electric vehicles and railway trains. Therefore, it’s very significant to develop the real-time fault diagnosis and detection systems for PMSM drive systems.

A PMSM drive system is usually embedded with at least two feedback current sensors, and faults in any of these sensors may lead to performance degradation [3], therefore, it is necessary to diagnose sensor faults to guarantee the safe and reliable operation of the drive system. Compared to sensor fault diagnosis of PMSM systems, there has been a lot of research on motor body faults or inverter faults [4,5,6,7,8,9,10]. Reference [11] proposed an offline method to diagnose current sensor faults for PMSM drive systems. In [12] a parity space approach based on redundancies in a temporal window to diagnose sensor faults was proposed, but the method only detected sudden faults. In [3] the authors proposed an Extended Kalman Filter method to diagnose current sensor faults for PMSM drive systems, but the performance would deteriorate at low speed, and it is not sensitive to slowly varying faults. A residual generation method based on an adaptive observer for phase current sensors was described in [13], but if the threshold is set larger, it will lead to misjudgments.

The technology of residual generation by means of a filter or observer for fault detection and isolation cannot directly estimate the failures, and this may cause problems such as omission and misjudgment if the threshold chosen is not suitable, so in practical engineering applications, fault diagnosis systems should have higher sensitivity to small and slow-variation faults, and at the same time, they should be robust to various uncertainties, so as to reduce the rates of false positives and missed fault reports. Therefore, this paper presents a current sensor fault reconstruction algorithm for an interior PMSM torque closed-loop drive system based on a sliding mode observer thanks to its better robustness to inaccurate mathematical models, external disturbances, and parameter perturbation [14,15]. First, current sensor faults are equated to actuator ones by a newly introduced state variable. Then, in αβ coordinates, based on the motor model with active flux linkage, a current sliding mode observer is constructed with specific equivalent control methodology to eliminate the effects of unknown disturbances on the system, and the phase current sensor faults are reconstructed by means of an adaptive method. Finally, an αβ axis current fault processing module is designed based on the reconstructed value. This method can accurately identify and reconstruct intermittent offset faults, slow-variation offset faults and abrupt gain faults in real-time, so the fault processing module can restrain the torque oscillation after current sensor fault occurs. The feasibility and effectiveness of the proposed method are verified by simulation and experimental tests on the RT-LAB platform.

## 2. IPMSM Mathematical Model

The stator voltage equations for the IPMSM in the rotating reference *dq* frame can be expressed as [16]:

(1) $$\left[\begin{array}{l} u_{d}\\ u_{q} \end{array}\right]=\left[\begin{array}{cc} R_{s}+DL_{d} & -\omega_{e}L_{q}\\ \omega_{e}L_{d} & R_{s}+DL_{q} \end{array}\right]\left[\begin{array}{l} i_{d}\\ i_{q} \end{array}\right]+\omega_{e}\left[\begin{array}{l} 0\\ \psi_{r} \end{array}\right]$$

The flux equations in the rotating reference *dq* frame are:

(2) $$\left\{ \begin{array}{l} \psi_{d}=L_{d}i_{d}+\psi_{r}\\ \psi_{q}=L_{q}i_{q} \end{array}\right.$$

The electromagnetic torque equation can be described as:

(3) $$T_{e}=\frac{3}{2}n_{p}[\psi_{r}+(L_{d}-L_{q})i_{d}]i_{q}$$

where *R_s_* stands for the stator resistance, *L_d_* and *L_q_* stand for the *dq* axis stator inductances, *u_d_*, *u_q_* and *i_d_*, *i_q_* are respectively the stator voltages and currents in the *dq* axis frame, *ψ_r_*, and *ψ_d_*, *ψ_q_* are respectively the permanent magnet flux linkage and the *dq* axis stator fluxes, *ω_e_* and *n_p_* are the electrical rotor speed and the number of pole pairs, respectively. *D* stands for the differential operator.

Through coordinate transformation, Equation (1) is transformed to be:

(4) $$\left[\begin{array}{l} u_{\alpha }\\ u_{\beta } \end{array}\right]=R_{s}\left[\begin{array}{l} i_{\alpha }\\ i_{\beta } \end{array}\right]+D\left[\begin{array}{cc} L_{1}+L_{2}\cos2\theta & L_{2}\sin2\theta \\ L_{2}\sin2\theta & L_{1}-L_{2}\cos2\theta \end{array}\right]\left[\begin{array}{l} i_{\alpha }\\ i_{\beta } \end{array}\right]+\omega_{e}\psi_{r}\left[\begin{array}{l} -\sin\theta \\ \cos\theta \end{array}\right]$$

where *L*_1_ = (*L_d_ + L_q_*)/2, *L*_2_ = (*L_d_ −* *L_q_*)/2, *u_α_*, *u_β_* and *i_α_*, *i_β_* are respectively the stator voltages and currents in the *αβ* axis frame, *θ* is the electrical position. Items with 2*θ* in Equation (4) show the salient features of IPMSM, and it is difficult for the estimation of the IPMSM status variable.

The voltage Equation (1) in the rotating reference dq frame can be reconstructed as:

(5) $$\left[\begin{array}{l} u_{d}\\ u_{q} \end{array}\right]=\left[\begin{array}{cc} R_{s}+DL_{q} & -\omega_{e}L_{q}\\ \omega_{e}L_{q} & R_{s}+DL_{q} \end{array}\right]\left[\begin{array}{l} i_{d}\\ i_{q} \end{array}\right]+\omega_{e}\left[\begin{array}{c} 0\\ \psi_{r}+\left(L_{d}-L_{q}\right)i_{d} \end{array}\right]+\left[\begin{array}{c} \left(L_{d}-L_{q}\right)Di_{d}\\ 0 \end{array}\right]$$

where $\left[\begin{array}{cc} R_{s}+DL_{q} & -\omega_{e}L_{q}\\ \omega_{e}L_{q} & R_{s}+DL_{q} \end{array}\right]$ is a symmetric matrix. It has nothing to do with the *d* axis stator inductance and eliminates the salient pole phenomenon of IPMSM. Through coordinate transformation, the Equation (5) in the stationary αβ-reference frame is transformed to be:

(6) $$\left[\begin{array}{l} u_{\alpha }\\ u_{\beta } \end{array}\right]=R_{s}\left[\begin{array}{l} i_{\alpha }\\ i_{\beta } \end{array}\right]+DL_{q}\left[\begin{array}{l} i_{\alpha }\\ i_{\beta } \end{array}\right]+\omega_{e}\left[\begin{array}{cc} \psi_{r}+(L_{d}-L_{q})i_{d} & 0\\ 0 & \psi_{r}+(L_{d}-L_{q})i_{d} \end{array}\right]\left[\begin{array}{l} -\sin\theta \\ \cos\theta \end{array}\right]$$

## 3. Sliding Mode Observers Design and Fault Reconstruction

According to Equation (6), one has:

(7) $$D\left[\begin{array}{l} i_{\alpha }\\ i_{\beta } \end{array}\right]=-\frac{R_{s}}{L_{q}}\left[\begin{array}{l} i_{\alpha }\\ i_{\beta } \end{array}\right]+\frac{1}{L_{q}}\left[\begin{array}{l} u_{\alpha }\\ u_{\beta } \end{array}\right]+\frac{\omega_{e}}{L_{q}}\left[\begin{array}{cc} \psi_{r}+(L_{d}-L_{q})i_{d} & 0\\ 0 & \psi_{r}+(L_{d}-L_{q})i_{d} \end{array}\right]\left[\begin{array}{l} -\sin\theta \\ \cos\theta \end{array}\right]$$

The active flux linkage is defined as [17,18]:

(8) $$\psi_{ext}=\psi_{r}+\left(L_{d}-L_{q}\right)i_{d}$$

Let *ψ_ext,__αβ_* represents the active flux linkage vector in the stationary *αβ-*reference frame, we have:

(9) $$\psi_{ext,\alpha \beta }=\left[\begin{array}{l} \psi_{ext,\alpha }\\ \psi_{ext,\beta } \end{array}\right]=\left[\begin{array}{cc} \psi_{r}+(L_{d}-L_{q})i_{d} & 0\\ 0 & \psi_{r}+(L_{d}-L_{q})i_{d} \end{array}\right]\left[\begin{array}{l} \cos\theta \\ \sin\theta \end{array}\right]$$

Let $x={\left[\begin{array}{cc} i_{\alpha } & i_{\beta } \end{array}\right]}^{T}$, $u={\left[\begin{array}{cc} u_{\alpha } & u_{\beta } \end{array}\right]}^{T}$, $A=\left[\begin{array}{cc} -\frac{R_{s}}{L_{q}} & 0\\ 0 & -\frac{R_{s}}{L_{q}} \end{array}\right]\;$, $B=\left[\begin{array}{cc} \frac{1}{L_{q}} & 0\\ 0 & \frac{1}{L_{q}} \end{array}\right]\;$, $C=\left[\begin{array}{cc} 1 & 0\\ 0 & 1 \end{array}\right]\;$, $F=\left[\begin{array}{cc} 0 & \frac{\omega_{e}}{L_{q}}\\ -\frac{\omega_{e}}{L_{q}} & 0 \end{array}\right]\;=-\frac{\omega_{e}}{L_{q}}J$, $J=\left[\begin{array}{cc} 0 & -1\\ 1 & 0 \end{array}\right]$.

According to Equation (7), the equation of PMSM model with sensor fault can be expressed as:

(10) $$\left\{ \begin{array}{l} \dot{x}(t)=Ax(t)+Bu(t)+F\psi_{ext,\alpha \beta }+Ed\\ y(t)=Cx(t)+Gf_{s} \end{array}\right.$$

where *f_s_* = [*f_sα_* *f_sβ_*]^T^ is the stator current sensor fault vector in the stationary *αβ-*reference frame, *d* = [*d_1_* *d_2_*]^T^ is the unknown disturbance vector, *d_1_* and *d_2_* are assumed to be bounded. “·” denotes the derivative. *x* is the state variable, *u* and *y* are respectively the input vector and output vector. $G=\left[\begin{array}{cc} 1 & 0\\ 0 & 1 \end{array}\right]\;$, $E=\left[\begin{array}{cc} 1 & 0\\ 0 & 1 \end{array}\right]\;$.

Consider a new state variable *z* which is a filtered version of *y*, and satisfies:

(11) $$\dot{z}=-az+by$$

where *a* and *b* are constant.

If we select a=0, b=1, Equation (11) can be rewritten as:

(12) $$\dot{z}=y=Cx(t)+Gf_{s}$$

According to Equations (10) and (12), a new system can be described as:

(13) $$\left\{ \begin{array}{l} \dot{x}(t)=Ax(t)+Bu(t)+F\psi_{ext,\alpha \beta }+Ed\\ \dot{z}=Cx(t)+Gf_{s}\\ w=z \end{array}\right.$$

where *w* is output vector of the new system. *f_s_* appears as an actuator fault in the new system and so the approach described earlier can be adopted.

The sliding mode observer can be designed as:

(14) $$\left\{ \begin{array}{l} \dot{\hat{x}}(t)=A\hat{x}(t)+Bu(t)+F\psi_{ext,\alpha \beta }+E\nu_{1}\\ \dot{\hat{z}}(t)=C\hat{x}(t)+G{\hat{f}}_{s}+\nu_{2} \end{array}\right.$$

where “^” denotes the estimated values. *v*_1_ and *v*_2_ are the sliding mode correction control signal.

(15) $$\nu_{1}=\left\{ \begin{array}{ll} L_{1}\frac{x-\hat{x}}{∥x-\hat{x}∥} & x-\hat{x}\neq 0\\ 0 & x-\hat{x}=0 \end{array}\right.$$

(16) $$\nu_{2}=\left\{ \begin{array}{ll} L_{2}\frac{z-\hat{z}}{∥z-\hat{z}∥} & z-\hat{z}\neq 0\\ 0 & z-\hat{z}=0 \end{array}\right.$$

where $L_{1}>0$ and $L_{2}>0$ are all to be designed.

The observer errors are defined as: $e_{x}=x-\hat{x}\;$, $e_{z}=z-\hat{z}$, $e_{s}=f_{s}-{\hat{f}}_{s}$.

The direct axis current component $i_{d}$ of the active flux linkage quickly converge to the given value $i_{d}^{*}$ when the bandwidth is high enough, this ensures the active flux linkage as a linear constant value [17,18]. The state estimation error equations can be expressed as:

(17) $${\dot{e}}_{x}=\dot{x}-\dot{\hat{x}}=Ae_{x}+E(d-\nu_{1})$$

(18) $${\dot{e}}_{z}=\dot{z}-\dot{\hat{z}}=Ce_{x}+Ge_{s}-\nu_{2}$$

Consider a Lyapunov function candidate:

(19) $$V=e_{x}^{T}e_{x}+e_{z}^{T}e_{z}+e_{s}^{T}Qe_{s}$$

where *Q* is a positive constant.

Differentiating the Lyapunov function and according to Equations (17) and (18), then:

(20) $$\begin{array}{ll} \dot{V} & ={\dot{e}}_{x}^{T}e_{x}+e_{x}^{T}{\dot{e}}_{x}+{\dot{e}}_{z}^{T}e_{z}+e_{z}^{T}{\dot{e}}_{z}+{\dot{e}}_{s}^{T}Qe_{s}+e_{s}^{T}Q{\dot{e}}_{s}\\ & ={\left[Ae_{x}+E(d-\nu_{1})\right]}^{T}e_{x}+e_{x}^{T}[Ae_{x}+E(d-\nu_{1})]+{\left(Ce_{x}+Ge_{s}-\nu_{2}\right)}^{T}e_{z}+e_{z}^{T}(Ce_{x}+Ge_{s}-\nu_{2})+2e_{s}^{T}Q{\dot{e}}_{s}\\ & =e_{x}^{T}Ae_{x}+{\left(d-\nu_{1}\right)}^{T}Ee_{x}+e_{x}^{T}Ae_{x}+e_{x}^{T}E(d-\nu_{1})+e_{x}^{T}Ce_{z}+e_{s}^{T}Ge_{z}-\nu_{2}^{T}e_{z}+e_{z}^{T}Ce_{x}+e_{z}^{T}Ge_{s}-e_{z}^{T}\nu_{2}+2e_{s}^{T}Q{\dot{e}}_{s}\\ & =2e_{x}^{T}Ae_{x}+2e_{x}^{T}E(d-\nu_{1})+2e_{z}^{T}Ce_{x}+2e_{s}^{T}Ge_{z}-2e_{z}^{T}\nu_{2}+2e_{s}^{T}Q{\dot{e}}_{s} \end{array}$$

Since *A* is a symmetric negative definite matrix, $C=\left[\begin{array}{cc} 1 & 0\\ 0 & 1 \end{array}\right]\;$, and $E=\left[\begin{array}{cc} 1 & 0\\ 0 & 1 \end{array}\right]\;$, then $\dot{V}$ can be expressed as:

(21) $$\begin{array}{ll} \dot{V} & \leq 2∥e_{x}∥∥E∥(∥d∥-L_{1})+2e_{z}^{T}Ce_{x}+2e_{s}^{T}Ge_{z}-2e_{z}^{T}\nu_{2}+2e_{s}^{T}Q{\dot{e}}_{s}\\ & \leq 2∥e_{x}∥∥E∥(∥d∥-L_{1})+2∥e_{z}∥(∥C∥∥e_{x}∥-L_{2})+2e_{s}^{T}(Ge_{z}+Q{\dot{e}}_{s})\\ & \leq 2∥e_{x}∥∥E∥(∥d∥-L_{1})+2∥e_{z}∥(∥C∥∥e_{x}∥-L_{2})+2e_{s}^{T}[Ge_{z}+Q({\dot{f}}_{s}-{\dot{\hat{f}}}_{s})] \end{array}$$

When the sensor gain changes very slowly as the time and environment change, or the sensor suddenly detects a break, power down or damage, the first derivative of the fault approximates to zero, that is ${\dot{f}}_{s}\approx 0$, then:

(22) $$\dot{V}\leq 2∥e_{x}∥∥E∥(∥d∥-L_{1})+2∥e_{z}∥(∥C∥∥e_{x}∥-L_{2})+2e_{s}^{T}(Ge_{z}-Q{\dot{\hat{f}}}_{s})$$

For the arbitrary initial conditions z(0), if the adaptive law algorithm for fault reconstruction is:

(23) $${\dot{\hat{f}}}_{s}=Q^{-1}Ge_{z}$$

Then:

(24) $$\dot{V}\leq 2∥e_{x}∥∥E∥(∥d∥-L_{1})+2∥e_{z}∥(∥C∥∥e_{x}∥-L_{2})$$

Select $L_{1}>∥d∥+\sigma_{1}$ and $L_{2}>C∥e_{x}∥+\sigma_{2}$, where $\sigma_{1}>0$, $\sigma_{2}>0$, then:

(25) $$\dot{V}\leq -2\sigma_{1}∥E∥∥e_{x}∥-2\sigma_{2}∥e_{z}∥\leq 0$$

From the Lyapunov theorem, the system will reach the sliding mode state in finite time.

Since *i_α_* and *i_β_* are practically calculated from *i_abc_*. In the case of only “a” and “b” phase current sensors, according to [13]:

(26) $$\left\{ \begin{array}{l} i_{\alpha }=i_{a}\\ i_{\beta }=\frac{i_{b}-i_{c}}{\sqrt{3}}=\frac{2i_{b}+i_{a}}{\sqrt{3}} \end{array}\right.$$

The effects of the *αβ* axis sensor faults *f_sα_* and *f_sβ_* are related to the errors in “a” and “b” phase sensor faults *f_a_* and *f_b_* as follows:

(27) $$\left\{ \begin{array}{l} f_{s\alpha }=f_{a}\\ f_{s\beta }=\frac{2f_{b}+f_{a}}{\sqrt{3}} \end{array}\right.$$

According to Equations (23) and (27), the adaptive law for the faults reconstruction in “a” and “b” phase sensors can be expressed as:

(28) $${\dot{\hat{f}}}_{a}={\dot{\hat{f}}}_{s\alpha }=Q^{-1}e_{z1}$$

(29) $${\dot{\hat{f}}}_{b}=\frac{\sqrt{3}{\dot{\hat{f}}}_{s\beta }-{\dot{\hat{f}}}_{s\alpha }}{2}=Q^{-1}\frac{\sqrt{3}e_{z2}-e_{z1}}{2}$$

## 4. Fault Diagnosis and Fault Processing

When no phase sensor faults occur, the fault reconstruction values are also zero. When sensor faults occur, the fault reconstruction values deviate from their zero value. The faults detection decision rule are expressed in Table 1, Where “1” stands for “sensor faults occur”, “0” stands for “no sensor faults occur”, “Flag i” represents “the fault flags”.

**Table 1:** Faults detection decision rule.

${\hat{f}}_{b}$	${\hat{f}}_{a}$	$Flag\;i_{b}$	$Flag\;i_{a}$
0	0	0	0
0	1	0	1
1	0	1	0
1	1	1	1

**Figure 1:**
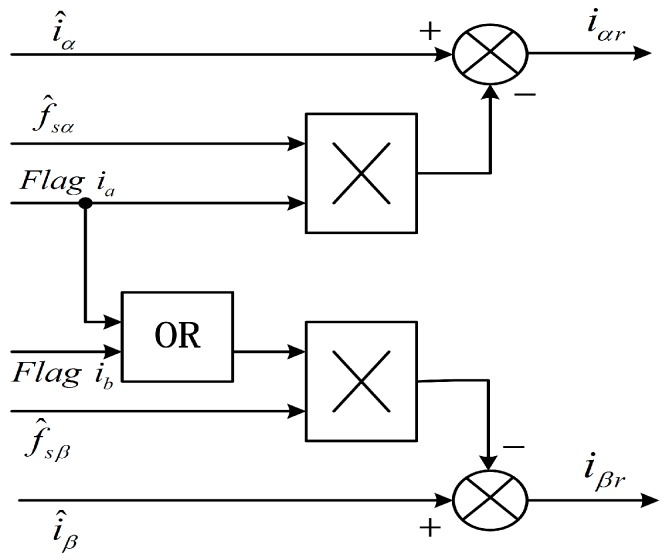
αβ axis current fault processing module.

After a sensor fault occurs, the torque oscillates since the original current balance is destroyed. The performance of the drive system will decrease. To achieve a performance similar to the pre-fault status, it is necessary to study the fault processing strategy. The αβ axis current fault processing module is shown in Figure 1. The fault processing equations can be expressed as:

(30) $$\left\{ \begin{array}{l} i_{\alpha r}={\hat{i}}_{\alpha }-{\hat{f}}_{s\alpha }\cdot Flag\;i_{a}\\ i_{\beta r}={\hat{i}}_{\beta }-{\hat{f}}_{s\beta }\cdot (Flag\;i_{a}⊕Flag\;i_{b}) \end{array}\right.$$

where $Flag\;i_{a}⊕Flag\;i_{b}$ is a logic AND operation equation, *i_ar_* and *i_br_* are the *αβ* axis feedback currents after fault processing. When no phase sensor faults occur, “Flag i” is zero value, the estimation of *αβ* axis current is adopted for the *αβ* axis feedback current. When sensor faults occur, “Flag i” equals one, the difference between the estimation of *αβ* axis current and its corresponding fault reconstruction is adopted for the *αβ* axis feedback current.

## 5. Simulations and Analysis

**Figure 2:**
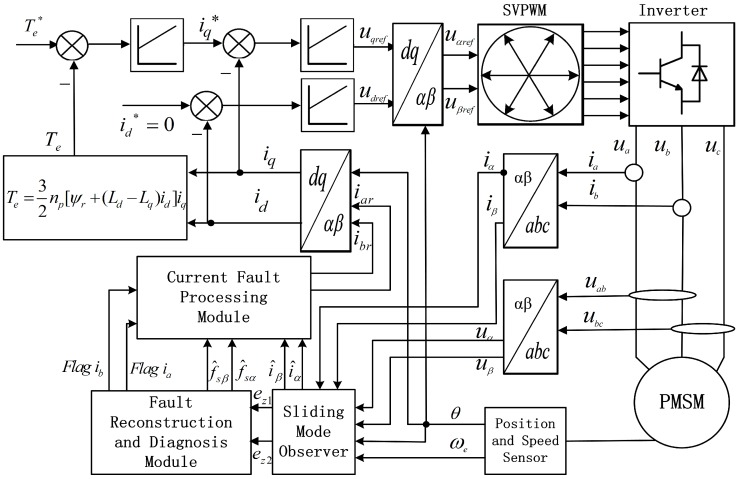
Block diagram of current sensor fault reconstruction and fault processing system for IPMSM.

The effectiveness of the fault reconstruction and fault processing method for PMSM drive system proposed in the paper was simulated experimentally. The block diagram of the simulation setup is shown in Figure 2.

The parameters for the interior PMSM of this study are tabulated in Table 2. The given speed and torque are 200 rad/s and 500 Nm, respectively. Let system unknown disturbances *d_1_* and *d_2_* be random noise, which value range is [−50 50]. To reduce chattering and eliminate high-frequency interference caused by the chattering, a successive approximation function can be used to substitute for the sign function sgn (·). Two cases are discussed:

**Table 2:** Interior PMSM parameters.

Quantity	Symbol	Value
Stator resistance	$R_{s}\;$	0.02 Ω
Q axis inductance	$L_{q}$	0.001500 H
D axis inductance	$L_{d}$	0.003572 H
Inertia	*J*	100 kg·m^2^
Magnetic flux	$\psi_{r}$	0.892 Wb
Number of pole pairs	*P*	4 pairs
Damping coefficient	*B*	0.001 Nm·s/rad
DC-bus voltage	$V_{dc}$	1500 V

(A) Intermittent offset fault on “b” phase sensor

The “b” phase sensor fault can be expressed as:

(31) $$f_{b}=\left\{ \begin{array}{cc} 0 & t<0.1s\\ -30 & 0.1s\leq t<0.3s\\ 0 & 0.3s\leq t<0.5s\\ -50 & 0.5s\leq t<0.7s\\ -20 & t\geq 0.7s \end{array}\right.$$

**Figure 3:**
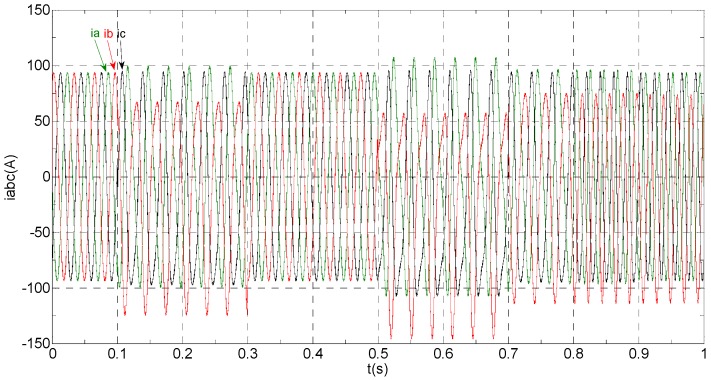
The measured value of the three-phase current.

**Figure 4:**
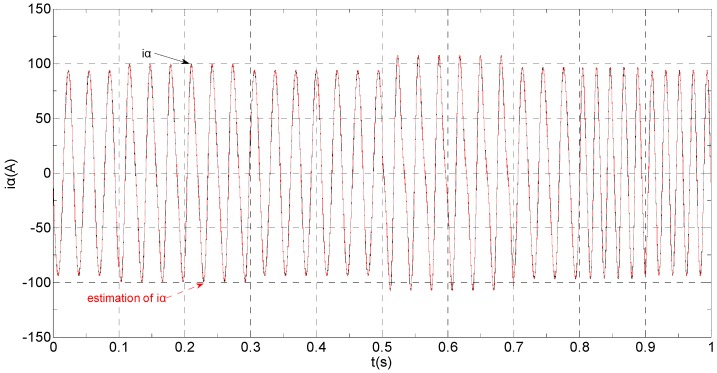
The actual and estimated value of *i_α_*.

**Figure 5:**
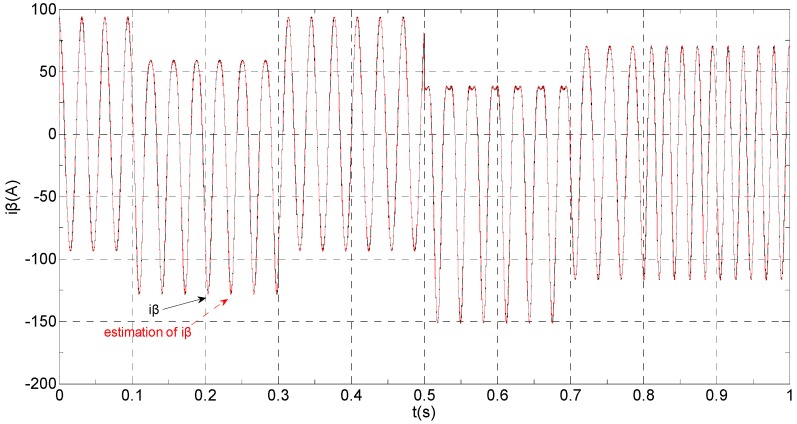
The actual and estimated value of *i_β_*.

**Figure 6:**
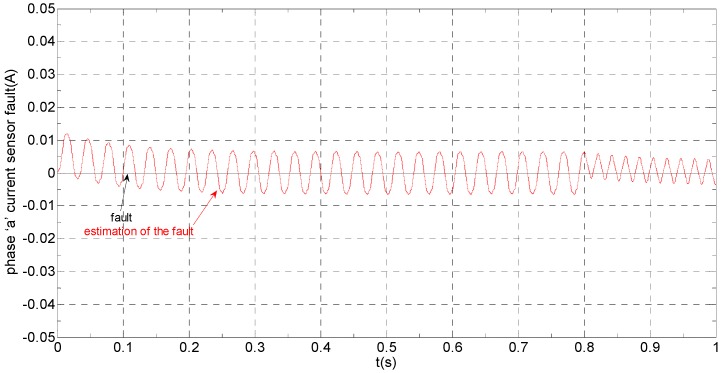
The current sensor fault and its reconstruction in phase “a”.

**Figure 7:**
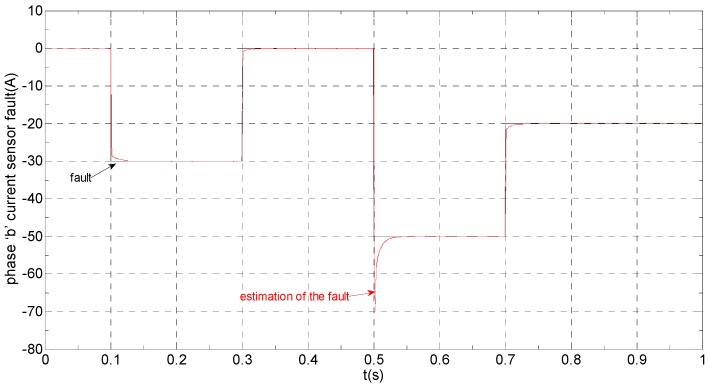
The current sensor fault and its reconstruction in phase “b”.

**Figure 8:**
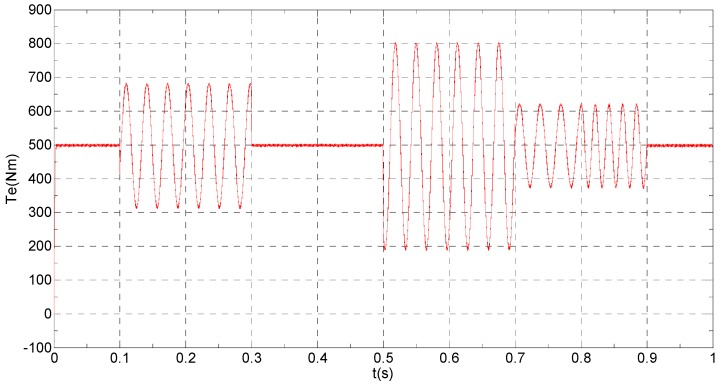
The electromagnetic torque.

The three-phase stator currents of the current sensor output are shown in Figure 3, and the actual and estimated values of *i_α_* and *i_β_* are shown in Figure 4 and Figure 5, respectively. The phase “a” and phase “b” current sensors fault reconstruction values are shown in Figure 6 and Figure 7, respectively. The electromagnetic torque is shown in Figure 8. As shown in the simulation figures, when an intermittent offset fault is added, the originally current balance is destroyed, the amplitudes of phase “a” and phase “c” currents increase slightly, the measured phase “b” sensor current produces an intermittent offset. The electromagnetic torque produces a corresponding equiamplitude oscillation. The adaptive law for fault reconstruction can accurately reconstruct the intermittent offset faults. When the fault value becomes zero at *t* = 0.3 s–0.5 s, the current and the torque are back to normal.

When the given speed increases to 300 rad/s at *t* = 0.8 s, the frequency of the current increases, accordingly, the equiamplitude oscillation frequency of the electromagnetic torque also increases, the change of the speed does not affect the observation of the current and the fault reconstruction. The current fault processing module is introduced at *t* = 0.9 s, the phase “b” and the *β* axis currents still experience the corresponding offset since the fault still exists, but the electromagnetic torque oscillation disappears and the torque value reaches the given value.

(B) Slow-variation offset fault on phase “a” sensor and abrupt gain fault on phase “b” sensor

The sensors faults can be expressed as:

(32) $$f_{a}=\left\{ \begin{array}{cc} 0 & t<0.3s\\ 100\tanh(t) & t\geq 0.3s \end{array}\right.$$

(33) $$f_{b}=\left\{ \begin{array}{cc} 0 & t<0.1s\\ 0.5i_{b} & t\geq 0.1s \end{array}\right.$$

**Figure 9:**
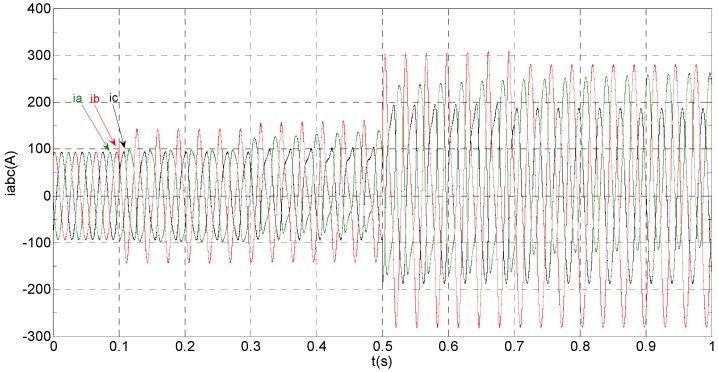
The measured value of three-phase current.

**Figure 10:**
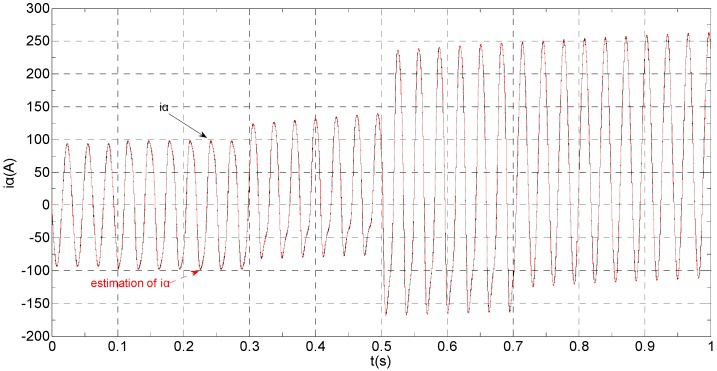
The actual and estimated value of *i_α_*.

**Figure 11:**
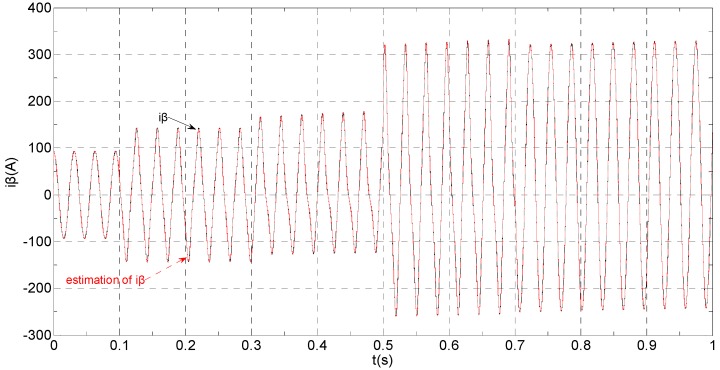
The actual and estimated values of *i_β_*.

**Figure 12:**
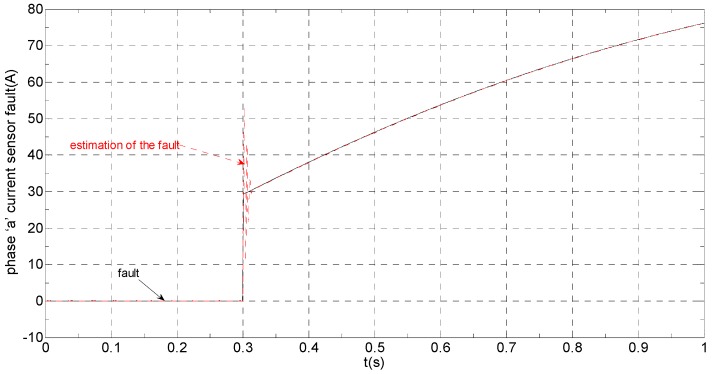
The current sensor fault and its reconstruction in phase “a”.

**Figure 13:**
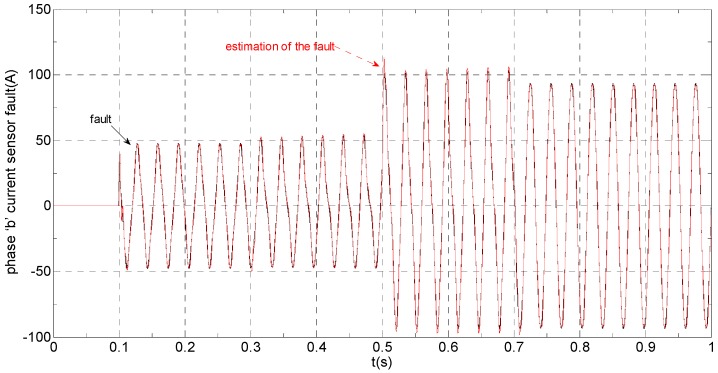
The current sensor fault and its reconstruction in phase “b”.

**Figure 14:**
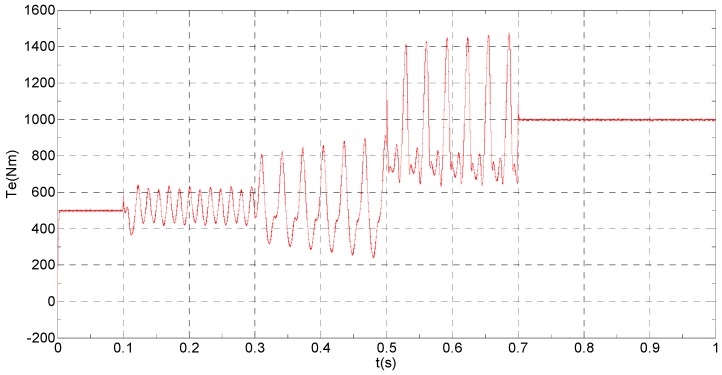
The electromagnetic torque.

The three-phase stator currents of the current sensor output are shown in Figure 9, and the actual and the estimated values of *i_α_* and *i_β_* are shown in Figure 10 and Figure 11, respectively. The phase “a” and phase “b” current sensors faults reconstruction values are shown in Figure 12 and Figure 13, respectively. The electromagnetic torque is shown in Figure 14. As shown in these figures, when the faults are added, the originally current balance is destroyed, the amplitudes of the *β* axis and phase “b” currents increase at *t* = 0.1 s and produce a slowly varying offset at *t* = 0.3 s, the measured phase “a” sensor current and the estimation of *α* axis current produce a slowly varying offset at *t* = 0.3 s. The gain sensor fault in phase “b” produces a corresponding equiamplitude torque oscillation at *t* = 0.1 s–0.3 s, The amplitude of the electromagnetic torque oscillation gradually increases when a slowly varying offset fault in the phase “a” sensor is introduced at *t* = 0.3 s whose initial value is about 29.17. The adaptive law for fault reconstruction can accurately reconstruct the slowly varying offset fault and abrupt gain fault. When the given torque increases to 1000 Nm at *t* = 0.5 s, the frequency of the current does not change and the amplitude of the current increases. The torque change does not affect the observation of the current and the fault reconstruction. The current fault processing module is introduced at *t* = 0.7 s, the three-phase currents and the *αβ* axis current are still in the corresponding fault status since the fault still exists, but the electromagnetic torque oscillation disappears and the torque value reaches 1000 Nm.

## 6. Hardware-in-the-Loop Experiments

**Figure 15:**
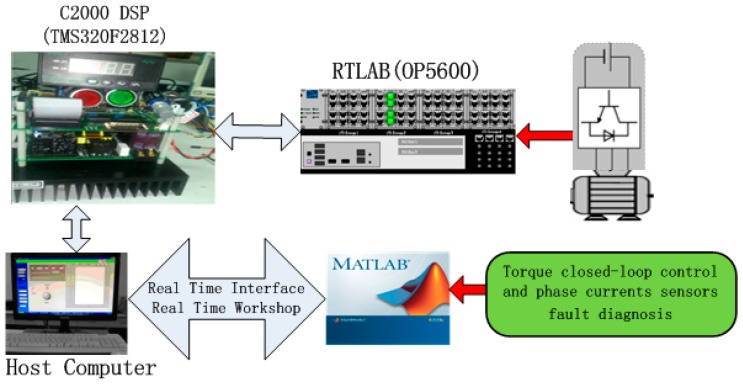
The configuration of RT-LAB experimental system.

**Figure 16:**
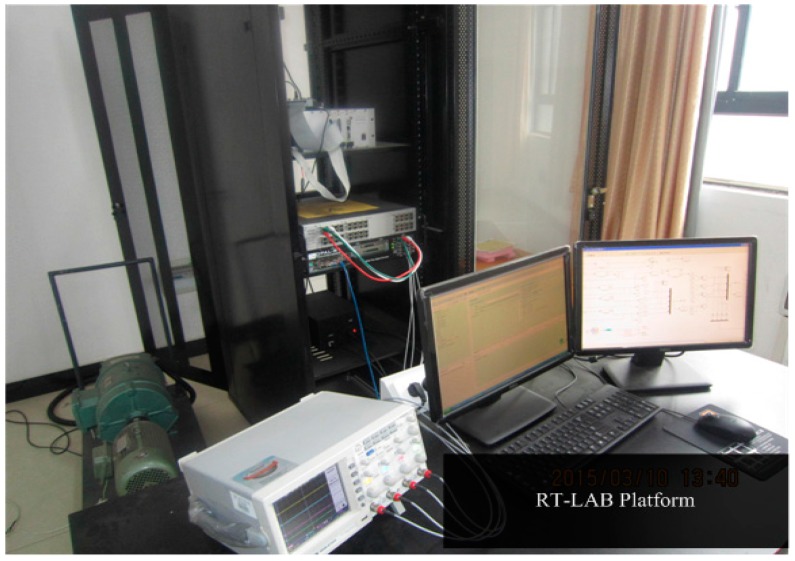
Photo of the RT-LAB experimental setup.

To verify the proposed algorithm, experiments have been carried out on a RT-LAB hardware-in-the-loop system. The configuration and platform are shown in Figure 15 and Figure 16, respectively.

**Figure 17:**
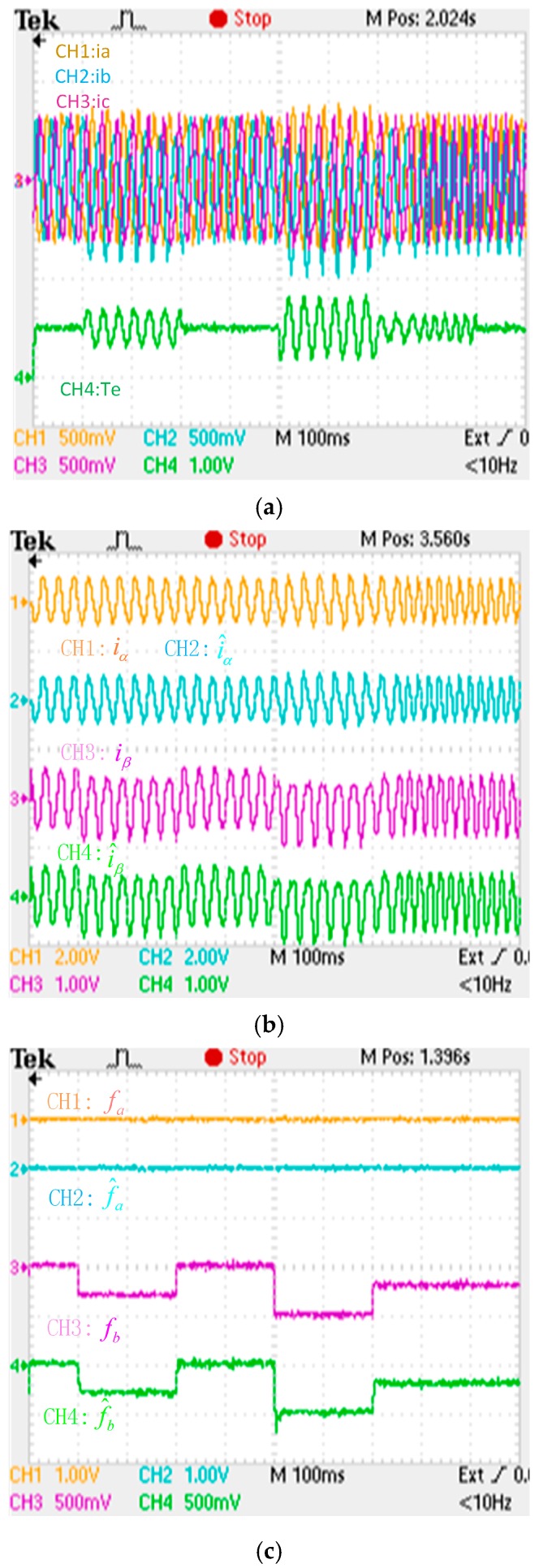
Experimental results when an intermittent fault is applied to the “b” phase current sensor. (**a**) phase currents and electromagnetic torque responses (iabc: 75 A/div; Te: 500 Nm/div; *t*: 100 ms/div); (**b**) actual and estimation *αβ* axis stator currents responses (iα: 200 A/div; iβ: 150 A/div; *t*: 100 ms/div); (**c**) phase currents fault and its reconstruction (fa: 1 A/div; fb: 50 A/div; *t*: 100 ms/div).

**Figure 18:**
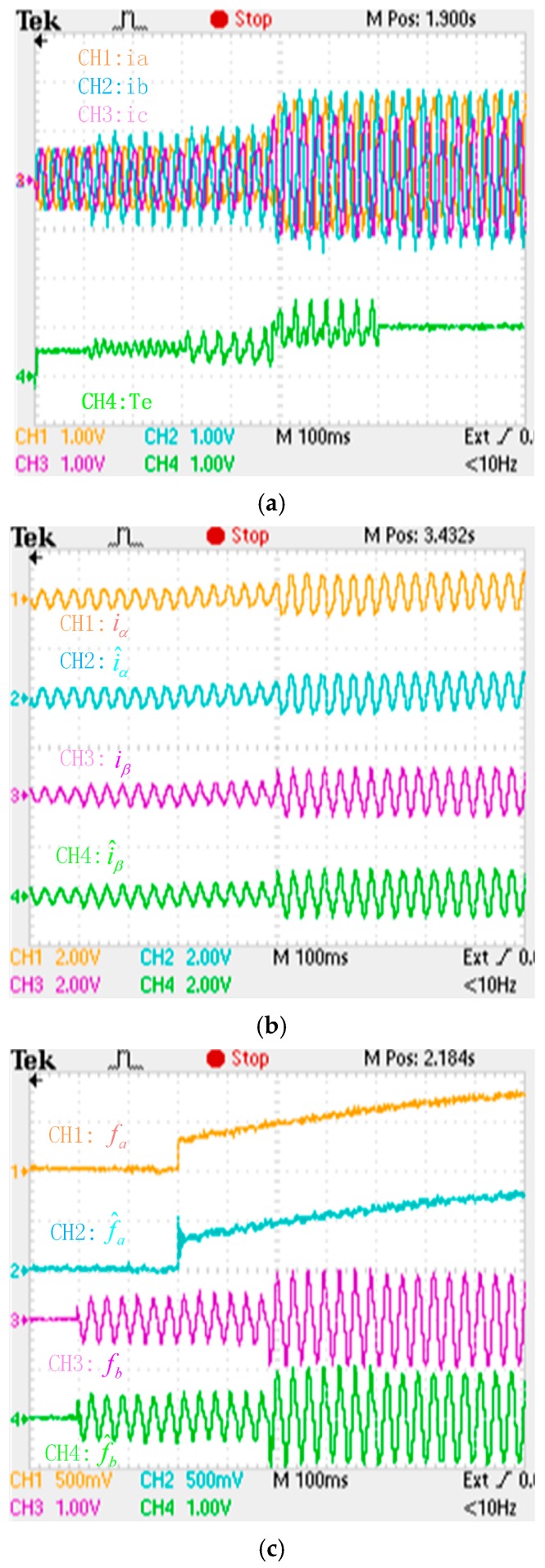
Experimental results when a slow-variation fault and a gain fault are respectively applied to the “a” phase current and “b” phase current sensors. (**a**) phase currents and electromagnetic torque (iabc: 150 A/div; Te: 1000 Nm/div; t: 100 ms/div); (**b**) actual and estimation αβ axis stator currents (iα: 500 A/div; iβ: 600 A/div; t: 100 ms/div); (**c**) phase currents fault and its reconstruction (fa: 50 A/div; fb: 100 A/div; t: 100 ms/div).

The controller is a TMS320F2812 DSP, The inverter and PMSM models use blocks from the RT-Events toolbox of RT-LAB OP5600. In this system, the PWM switching frequency is set at 5 KHz. The sampling period is chosen as 20 μs. The experimental results are shown in Figure 17 and Figure 18.

Through comparison with the simulation results, the adaptive law of the fault reconstruction can effectively reconstruct intermittent offset faults, slowly varying offset faults and abrupt gain faults of the current sensor, and the αβ axis current fault processing module can restrain the torque oscillation after a fault occurs, and the drive system has better dynamic and higher real-time performance.

## 7. Conclusions

This paper presents a phase current sensor fault reconstruction method for an interior PMSM torque closed-loop drive system based on a sliding mode observer. The motor model with active flux linkage is constructed first. Next a sliding mode current observer is designed in αβ coordinates to eliminate the effects of unknown disturbances by using a specific equivalent control methodology. Then, the phase current sensor faults are reconstructed by means of an adaptive method. Finally, an αβ axis current fault processing module is designed based on the reconstructed value. This method can accurately identify and reconstruct intermittent offset faults, slowly varying offset faults and abrupt gain faults in real-time, and the αβ axis current fault processing module can restrain the torque oscillation of the system after a fault occurs. The feasibility and effectiveness of the proposed method are verified by simulation and experimental tests on the RT-LAB platform.
